# Biologic Agents and Disease Emergence

**DOI:** 10.3201/eid1007.AC1007

**Published:** 2004-07

**Authors:** Polyxeni Potter

**Affiliations:** * Centers for Disease Control and Prevention, Atlanta, Georgia, USA

**Keywords:** biologic agents, disease emergence, cover text, Jaune Quick-to-See Smith, Rain

**Figure:**
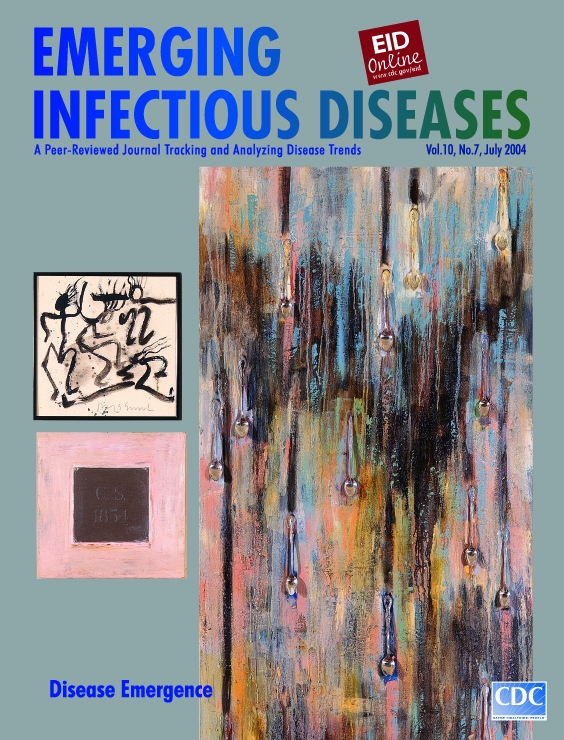
Jaune Quick-To-See Smith, Enrolled Flathead Salish (b. 1940), Rain (1990) Mixed media 203.2 cm x 76.2 cm, 30.48 cm x 30.48 cm, 30.48 cm x 30.48 cm. Fine Art Collection, Heard Museum, Phoenix, Arizona, USA

"I think of my work as an inhabited landscape, never static or empty…. The wind ruffles; ants crawl; a rabbit burrows" (*1*). A painter of Salish, French-Cree, and Shoshone heritage, Jaune Quick-to-See Smith was born in St. Ignatius, Montana, and raised on the Flathead Reservation. She studied art at the University of New Mexico, but her personal aesthetic and her poetic association with nature go back to her childhood, when under her father's tutelage, she learned "to see and feel" (*2*).

Feeling and astute observation characterize Smith's representational, abstract, and symbolic landscapes (*3*). A prolific artist, inspired by the formal innovations of Pablo Picasso, Paul Klee, and others, she uses paint, collage, and other media to compose unique forms on tactile surfaces that explore the continuum of life, the connection between living beings and the land, and the fundamental relationship between all things. As artist, curator, and lecturer, she has promoted understanding and reverence of nature, articulated Native aesthetic tradition in a modern art context, and made her mark on the contemporary American art scene.

Native American cultures did not refer to art as a separate discipline before the mid-19th century. Cultural materials with aesthetic value (totem poles, pottery, beadwork) were integrated into everyday life and traditional practices. Artists painted geometric patterns or symbolic representations of figures on readily available media (sand, hides, clay); wood, bone, and stone were carved too for a three-dimensional effect. The iconography and function of the work varied widely with region and tribe, but all objects were imbued with spirituality and were meant to serve the community (*4*).

Throughout the 20th century, Native American work was influenced by European realism. The early years were dominated by depictions of ceremonial dances and genre scenes painted in linear, decorative style. Later years saw a multiplicity of styles, including pop art and art deco (*4*). Smith, like many contemporary artists rooted in Native traditions, uses unique forms and nonfigurative visual language to express truths at once deeply personal and profoundly universal.

Rain, on the cover of this month's Emerging Infectious Diseases, comprises three parts in a nontraditional ensemble depicting one of Smith's favored themes, the close bond between humanity and nature. The main part of the ensemble is a long visual field painted in somber tones and punctuated with glistening metal spoons, arranged without regularity but with internal vertical symmetry, synchronized as they are by a powerful guiding force, gravity. The colors (grays, yellows, browns, reds) smear and run, blending into each other in alternating flat patches and dark grooves that give the strands of runny paint a three-dimensional effect.

In this abstract but anthropomorphic image of nature, without painting a single human face, Smith captures centuries of sorrow. As if long held in by a natural wall, massive sadness finally seeps through, forming large quiet drops. Emotions of every hue caused by every possible offense meet in a discolored torrent of grievance. Darkness, pain, and injustice meet grime, ignorance, arrogance, and greed. The inner human pain touches the outer pain of nature, dissolving the canvas in a cathartic rain of tears.

The work, which delivers a compelling ecologic and cultural message, has two other parts. To the side of the main canvas is a black-and-white sketch of fluid, stylized figures engaged in a free-form dance. Below the framed dancers is a silver-and-pink panel whose center is engraved with the initials "C.S." for Chief Seattle, Suquamish (1786–1866) and an eloquent advocate of the earth.

Smith's icon of suffering engages the viewer in an empathetic recall of past wrongs, from environmental degradation to cultural annihilation through, among other causes, the spread of disease. When smallpox was introduced on the North American continent, it devastated the immunologically naïve Native population. Later, the human spirit, whose survival is at the heart of 20th century art (*5*), triumphed over the disease, eradicating it from the planet. Challenges to the bond between humanity and nature continue, emerging unpredictably and without end. Anthrax spores, fully understood and refined, were released into the U.S. postal system, reviving the specter of intentional biologic contamination. Like scientific efforts to anticipate and curtail the threat (*6*), Smith's work confronts the pain of human and environmental catastrophe, embracing efforts to prevent it.
